# A neonate with intrauterine growth restriction and pseudo‐Bartter syndrome due to severe maternal eating disorder: A case report

**DOI:** 10.1002/ccr3.3223

**Published:** 2020-08-06

**Authors:** Evgeniya Babatseva, Ilias Chatziioannidis, Alexia ‐ Angeliki Tagaraki, Despoina Tramma, Kalliopi Dampala, Efthymia Chatzitoliou, Efthymia Papacharalambous, Georgios Mitsiakos, Christos Tsakalidis, Paraskevi Karagianni, Maria Lithoxopoulou, Kleanthis Anastasiadis, Vasiliki Soubasi

**Affiliations:** ^1^ 2nd Department of Neonatology and NICU Aristotle University of Thessaloniki “Papageorgiou” Hospital Thessaloniki Greece; ^2^ 4th Department of Pediatrics Aristotle University of Thessaloniki “Papageorgiou” Hospital Thessaloniki Greece; ^3^ 1st Department of Obstetrics and Gynecology Aristotle University of Thessaloniki “Papageorgiou” Hospital Thessaloniki Greece; ^4^ 2nd Department of Pediatric Surgery Aristotle University of Thessaloniki “Papageorgiou” Hospital Thessaloniki Greece

**Keywords:** eating disorders, hypokalemia, intrauterine growth restriction, metabolic alkalosis, pseudo‐Bartter syndrome

## Abstract

Maternal diet before and during pregnancy plays an important role for the developing fetus. Any eating disorder in this period can cause transient or/and permanent negative effects on the mother and her offspring.

## Graphical abstract

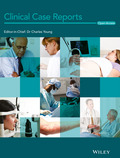

## 1 INTRODUCTION

Pseudo‐Bartter syndrome, a rare disorder, similar to Bartter syndrome, lacks renal tubular pathology. We present the case of a term small for gestational age neonate with metabolic alkalosis since birth with gradual decline within first week of life. Similar laboratory findings were observed in baby's mother, suffered from severe eating disorders.

Pseudo‐Bartter syndrome is a rare disorder caused by chloride poor diet, loss of sodium or chloride in the urine/stool, and vomit or prostaglandin infusion.^1^, ^2^ It is characterized by hypokalemic, hypochloremic metabolic alkalosis. Other findings include hyponatremia, high levels of renin and aldosterone, normal blood pressure, and hyperplasia of the juxtaglomerular apparatus.^3^, ^4^, ^5^ Anorexia nervosa (AN) and bulimia nervosa (BN) are severe eating disorders characterized by serious behavioral, psychological, and physiological disturbances. ΑΝ/ΒΝ during pregnancy affects not only the mother but also her fetus. These conditions can cause perinatal mortality, prematurity, and fetal outcomes as small and large for gestational age births, intrauterine growth restriction, neurobehavioural impairment but also acid‐base and electrolyte imbalance.^4^, ^6^, ^7^, ^8^

We report a neonate with severe intrauterine growth restriction pattern whose mother suffered severe AN intensified during pregnancy with coexistent pseudo‐Bartter syndrome attributed to her long‐term eating disorder.

## 2 CASE PRESENTATION

The patient, a term male at 37 weeks’ gestational age born from a primigravida 26‐year‐old mother, was admitted to our NICU soon after birth for presenting signs of respiratory distress and severe intrauterine restriction.

Prenatal screening of third trimester despite normal amniotic fluid revealed that the fetus was suffering intrauterine growth restriction. The newborn was a symmetrical, small for gestational age (SGA) with birth weight 2285 gr (P 3rd‐10th), length 44 cm (P 3rd), and head circumference 32 cm (P 3rd‐10th). He was born with cesarean section, due to fetal distress with Apgar score of 7 and 8 at 1 and 5 minutes, respectively. Maternal history revealed depression and anorexia nervosa since 18 years of age. AN was intensified during the second and third trimester of pregnancy. Mother's body weight at first and third trimester was 52 and 43 kg, respectively; with a height of 162 cm BMI fell from 19, 8 kg/m^2^ to 16, 3 kg/m^2^. An arterial blood analysis from the umbilical cord within normal range according to his gestational age and day of life.^9^ Maternal showed metabolic alkalosis, hypokalemia, hypochloremia, and hyponatremia, which were also confirmed in subsequent measurements. It was confirmed that the infant had no episodes of vomiting, diarrhea/multiple stools and was not on any drug administration. Urine analysis for hypercalciuria, chloride concentration, and ultrasonography of the kidneys were normal. In view of the intrauterine growth restriction, the infant was evaluated for intrauterine infection that had normal findings. The arterial blood pressure of the baby was blood analysis revealed similar findings (Table 1). Total parenteral nutrition started on 1st day of life (dol), while enteral nutrition on 2nd dol with electrolyte supplementation. Alkalemia and electrolyte disorders were progressively corrected within the first week of life. The newborn had no signs of respiratory distress after 12 hours admission in NICU, and with an uneventful NICU hospitalization, he was finally discharged at 10 dol.

**Table 1:** Maternal and neonatal blood gas analysis at 1st dol

	UC	Neonate	Mother
pH	7.67	7.41	7.61
pCO_2_ (mmHg)	34.9	63.1	34.1
pO_2_ (mmHg)	32.2	53.0	270.6
HCO_3_ (mmol/L)	38.9	38.7	33.3
Base excess (mmol/L)	17.2	10.7	11.0
Sodium (mmol/L)	122.2	126.3	125.8
Potassium (mmol/L)	2.8	2.59	1.69
Calcium (mmol/L)	1.04	1.17	0.92
Chloride (mmol/L)	87	81	88
Lactate (mmol/L)	1.21	2.02	1.56
Glycose (mg/dL)	68	120	79

Abbreviation: UC, Umbilical Cord.

At 6 months of life, the baby had a normal physical and neurolodevelopmental examination.

## 3 DISCUSSION

Balanced maternal nutrition before and during pregnancy is a key factor to the fetal well‐being and normal intrauterine growth.^7^ Eating disorders in pregnancy could have negative physical and mental impact on the mother and her offspring during perinatal and postnatal period.^4^ Metabolic alkalosis in the first few days of life is extremely rare and requires a detailed history of the mother before and during pregnancy. In most cases, it is characterized by the absence of primary kidney damage and is of transient character.^10^ It may be due to renal, gastrointestinal, metabolic, endocrine causes, or drug exposure. Additionally, low potassium levels should be considered as a potential dangerous electrolyte disturbance.^4^

Maternal metabolic and electrolyte disorders similar to her offspring are characteristic of pseudo‐Bartter syndrome.^11^ Bartter syndrome (BS) is a rare hereditary renal tubular disorder that affects around 1 in 1 000 000 of the population. This genetic defect is linked to mutations in genes that encode sodium/potassium/chloride transporters.^1^ These are located in the loop of Henle and regulate extracellular volume and electrolyte balance affecting body fluids composition.^1^ Common findings of hereditary BS are polyhydramnios, premature delivery, growth retardation of the neonate with polyuria, and normal blood pressure.^1^, ^4^

In pseudo‐Bartter syndrome, biochemical abnormalities are identical to Bartter syndrome, without the genetic background. A variety of factors, such as long‐term administration of diuretics or prostaglandin E, persistent vomiting or diarrhea, a chronic chloride‐deficient diet, laxative abuse, and cystic fibrosis, are implicated in pseudo‐Barter's pathophysiology.^1^, ^10^, ^12^, ^13^ Numerous cases of pseudo‐Bartter syndrome in neonates, caused by congenital chloride diarrhea, prostaglandin E administration, and persistent vomiting have been described in the literature.^5^, ^8^, ^14^

Maternal eating disorders (AN, BN, binge eating disorder [BED], other specified feeding and eating disorders [OSFED]) during pregnancy, are directly linked to acid‐base imbalance and also have a higher risk for fetal/neonatal complications compared with malnourished pregnant women who are symptom free.^6^, ^15^ Kimmel et al in their review pointed that eating disorders associate with serious gynecologic problems as unplanned pregnancy, higher risk for cesarean section, infertility, miscarriage, postpartum depression, and sexual dysfunction.^16^ Maternal weight loss secondary to an eating disorder or hyperemesis gravidarum is associated to intrauterine growth retardation.^10^, ^17^, ^18^ Active AN associates to lower placental weight, symmetric growth restriction, and newborns with lower head circumference/size as this was our case.^17^, ^18^

Intrauterine growth restriction predisposes to tubular injury and long‐term renal damage with mechanisms involved such as chronic in utero hypoxia and reduced number of nephrons.^19^ Higuchi et al reported a total of 4 cases out of 153 low birth weight infants with pseudo‐Bartter syndrome, one being small for gestational age out of two having intrauterine growth restriction.^10^ Also, Mathot et al published a similar case of a late preterm, appropriate for his gestational age neonate born from a mother with AN and coexistent pseudo‐Bartter syndrome.^8^

Maternal history of severe AN, associated fetal intrauterine growth restriction, coexistent and transient metabolic alkalosis, and electrolyte disturbances in both mother and infant differentiates this case from Bartter syndrome.

## 4 CONCLUSION

A rare case of metabolic alkalosis early in life should bear in mind that the primary cause may not be fetal/neonatal but of maternal origin. A detailed maternal history for active eating disorders may reveal asymptomatic pseudo‐Bartter syndrome. Finally, a close monitoring of these women during pregnancy will contribute to proper management, to ensure prevention of complications, both maternal and neonatal.

## CONFLICT OF INTEREST

None declared.

## ETHICAL APPROVAL

Obtained.
